# Case Report: Negative pressure wound therapy with instillation and dwell time as adjuvant therapy for limb salvage in a complicated necrotizing fasciitis on ischemic diabetic foot

**DOI:** 10.3389/fsurg.2026.1687275

**Published:** 2026-02-05

**Authors:** Shiuan-Fu Chen, Chang-Cheng Chang

**Affiliations:** 1 Division of General Practice, Department of Medical Education, Changhua Christian Hospital, Changhua, Taiwan; 2 School of Medicine, College of Medicine, China Medical University, Taichung, Taiwan; 3 Department of Cosmeceutics, China Medical University, Taichung, Taiwan; 4 Division of Plastic and Reconstructive Surgery, Department of Surgery, China Medical University Hospital, Taichung, Taiwan; 5 Aesthetic Medical Center, China Medical University Hospital, Taichung, Taiwan

**Keywords:** dermal substitute, diabetic foot ulcer, limb salvage, multidisciplinary management, multimodal therapy, necrotizing fasciitis, negative-pressure wound therapy with instillation and dwell time, negative-pressure wound therapy

## Abstract

**Background:**

Necrotizing fasciitis superimposed on ischemic diabetic foot ulcers represents a complex and limb-threatening condition, especially in patients with multiple comorbidities such as end-stage renal disease, peripheral arterial occlusive disease, and poorly controlled diabetes mellitus. Optimal wound management strategies that minimize surgical burden while enhancing infection control and tissue regeneration are essential. This case highlights the use of multimodal adjuvant therapies and multidisciplinary management for limb salvage in a high-risk patient.

**Case presentation:**

A 60-year-old woman with a Wagner grade 4 ischemic diabetic foot ulcer complicated by deep invasive necrotizing fasciitis underwent urgent extensive debridement, fasciotomy, and sequestrectomy, resulting in a large soft tissue defect with substantial bone and tendon exposure.

**Management:**

A staged wound management approach was adopted, beginning with negative pressure wound therapy with instillation and dwell time using normal saline to promote bioburden reduction and granulation tissue formation. This was followed by the application of a dermal substitute combined with conventional negative pressure wound therapy to support dermal regeneration. Throughout the treatment course, multidisciplinary care was provided to optimize the management of systemic comorbidities.

**Outcome:**

After four cycles of negative pressure wound therapy with instillation and dwell time, more than 70 percent of the wound bed, including previously exposed bone and tendon areas, was covered with healthy granulation tissue. Over the following three months, further tissue regeneration was achieved with the dermal substitute and negative pressure wound therapy. No additional surgical debridement was required, and limb preservation was successfully maintained without progression to systemic sepsis. Functionally, the patient ambulated with mobility assistance and reported minimal pain with good treatment acceptability. The patient subsequently died at month 5 from cardiovascular disease unrelated to the wound; wound stability through month 5 was recorded only in treating-team documentation.

**Conclusion:**

This case demonstrates that a carefully staged multimodal adjuvant therapy protocol, incorporating negative pressure wound therapy with instillation and dwell time, dermal substitute application with negative pressure wound therapy, and multidisciplinary management of comorbidities, can serve as an effective limb salvage strategy in high-risk patients with diabetic foot ulcer-associated necrotizing fasciitis and extensive soft tissue defects when conventional reconstructive options are not feasible. This multimodal protocol may inform practice in high-risk or resource-limited settings.

## Introduction

Diabetes mellitus is a growing global health concern, with 589 million adults affected in 2024 and projections reaching 853 million by 2050 (1). Among its complications, diabetic foot ulcers (DFUs) are particularly debilitating, affecting 19%–34% of diabetic patients over their lifetime and often progressing to infection, gangrene, necrotizing fasciitis (NF), or osteomyelitis if not properly managed (2). Diabetes-related foot infections occur in up to 50% of cases (3). Given the high burden of DFU-related complications, timely and effective management is critical, especially in patients with multiple comorbidities.

NF is a rare but fatal soft-tissue infection characterized by rapid progression, and delays in recognition or surgical debridement are strongly associated with sepsis, major amputation, and death. Clinical signs include erythema, ecchymosis, progressive pain, fever, and in advanced cases, bullae or necrosis. Diagnosis is supported by a Laboratory Risk Indicator for Necrotizing Fasciitis (LRINEC) score ≥6 and imaging findings such as fascial thickening, gas formation, or inflammation (4–7). NF occurs in approximately 4%–7% of DFU patients, often in the setting of significant comorbidities including cardiovascular, renal, or hepatic dysfunction (8–10).

NF-related complications such as sepsis, organ failure, limb loss, or death result in mortality rates approaching 22%–29% (8, 10). Standard treatment requires urgent debridement or fasciotomy, often resulting in extensive soft tissue defects with exposed bone or tendon. In such high-risk wounds, adjuvant therapies play a critical role in enhancing infection control, promoting granulation, and reducing the need for major reconstruction or amputation.

Adjuvant therapies for complex or infected diabetic foot wounds include negative pressure wound therapy (NPWT) and dermal substitutes, both of which have demonstrated clinical efficacy and safety (11, 12). NPWT with instillation and dwell time (NPWTi-d) further enhances bioburden control and granulation in chronic or infected wounds (13). However, current evidence supporting the use of NPWTi-d or dermal substitutes in postoperative DFU-associated NF remains limited to case reports and small series (14–18). Importantly, no studies have explored the combined use of NPWTi-d and dermal substitutes with NPWT in wounds with extensive bone and tendon exposure.

We present the case of a 60-year-old woman with DFU-associated NF and multiple comorbidities who developed a large post-debridement defect with substantial tendon and bone exposure. A staged, multimodal strategy using NPWTi-d followed by dermal substitute with NPWT, supported by multidisciplinary care, achieved successful wound closure and limb preservation without the need for further reconstruction or amputation.

## Case presentation

A 60-year-old female patient presented with a history of diabetes mellitus, hypertension, hyperlipidemia, hyperuricemia, and three-vessel coronary artery disease, all of which were managed with oral medications. She had a significant vascular history of peripheral arterial occlusive disease (PAOD), with prior stenting of both superficial femoral arteries and balloon angioplasty of the right popliteal artery, and three years later developed in-stent restenosis requiring repeat percutaneous transluminal angioplasty and additional stenting. Furthermore, she also had end-stage renal disease (ESRD) on hemodialysis, a 20 pack-year smoking history, and overweight.

**Figure 1:**
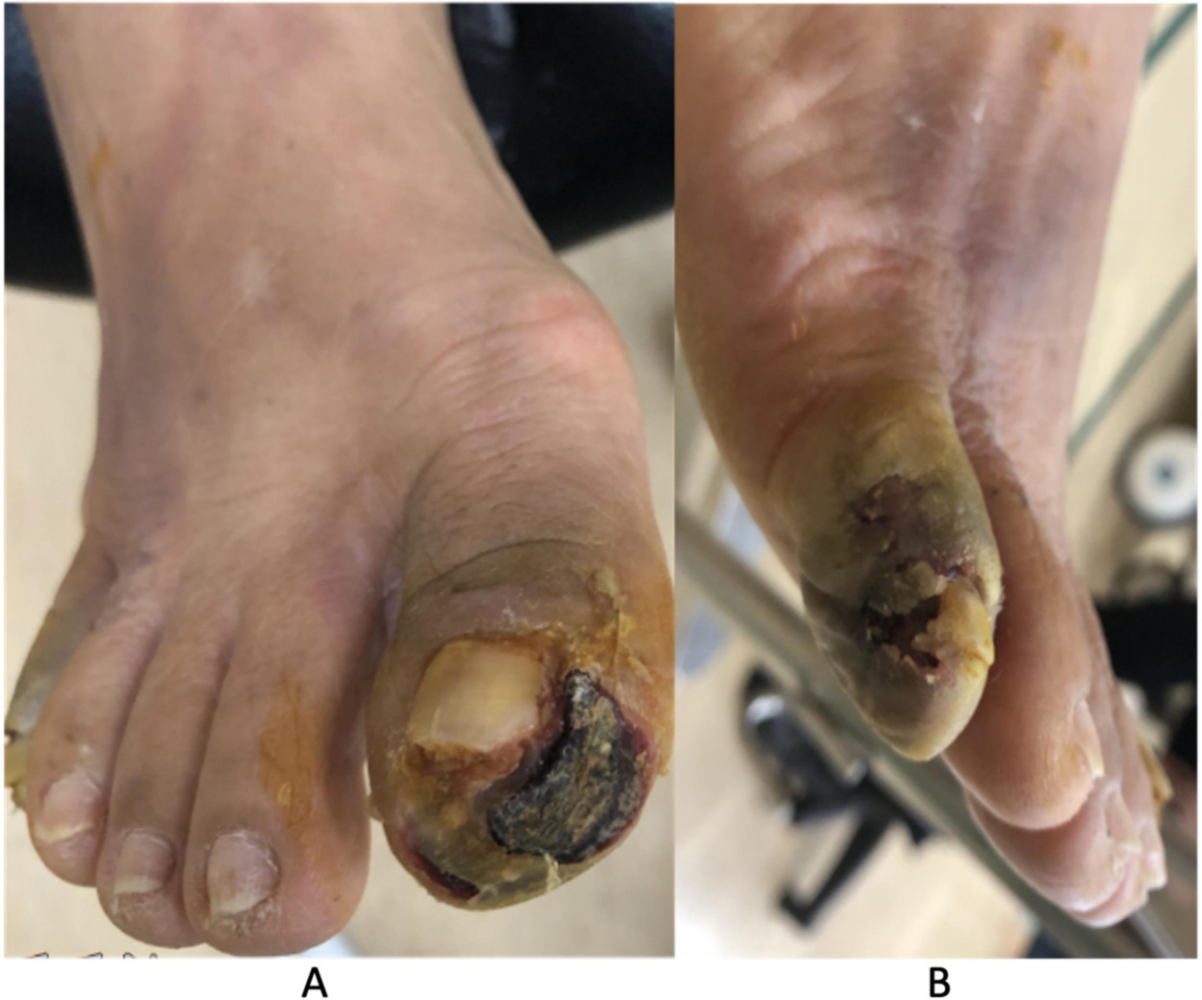
Clinical presentation of DFUs involving the right foot. **(A)** Full-thickness necrotic ulceration with black eschar and surrounding erythema over the right hallux. **(B)** Gangrenous changes and soft tissue necrosis at the right fifth toe, consistent with Wagner grade 4 and University of Texas Wound Classification System stage D3 lesions.

The patient developed progressive DFUs affecting the first and fifth toes of the right foot, presenting with malodor, gangrene, and localized tenderness with cyanosis. The DFUs were classified as Wagner grade 4 and University of Texas Wound Classification System stage D3, prompting amputation of the first and fifth toes (Figures 1A,B). However, similar changes later developed in the remaining toes of the right foot, leading to additional toe amputations.

Approximately one year later, the patient presented with acute pain, swelling, erythema, warmth, and rapidly progressive necrosis over the dorsum of the right foot. Physical examination revealed a 7 × 11 cm erythematous necrotic plaque with marked tenderness. Laboratory evaluation (Supplementary Table S1) yielded a LRINEC score of 12. Imaging revealed soft tissue swelling, intra-articular air, and diffuse vascular calcification, and the ankle–brachial index was 0.47 on the right and 0.70 on the left, consistent with a diagnosis of necrotizing fasciitis in the context of peripheral arterial occlusive disease (PAOD).

**Figure 2:**
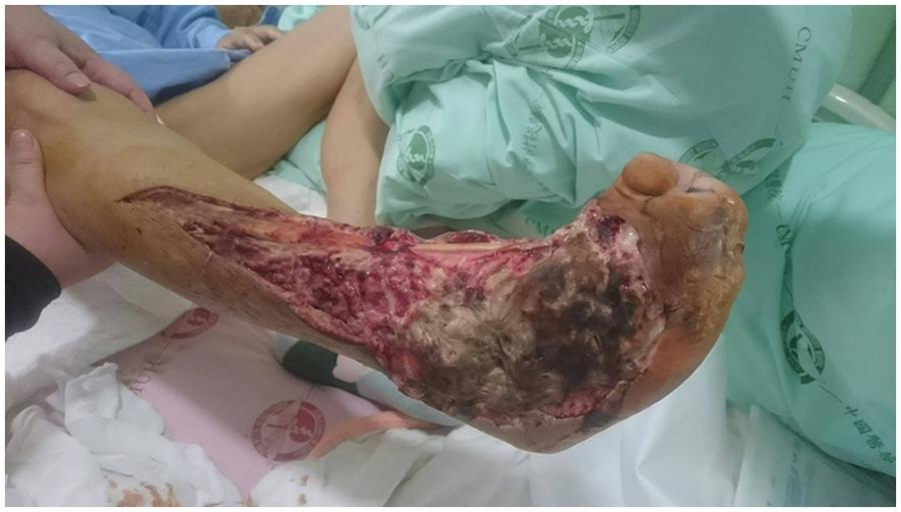
Clinical appearance of the right foot after extensive debridement, fasciotomy, and sequestrectomy. The wound measured approximately 250 cm^2^ (about 3% of total body surface area) of soft tissue defect with 95 cm^2^ area of bone exposure and significant tendon exposure.

Empirical broad-spectrum intravenous antibiotics including teicoplanin, piperacillin-tazobactam, and clindamycin were initiated. Given the patient's comorbidities and an American Society of Anesthesiologists physical status >3, amputation was considered. However, due to the patient's preference for limb preservation, urgent debridement, fasciotomy, and sequestrectomy were performed, resulting in a 250 cm^2^ soft tissue defect with 95 cm^2^ bone and tendon exposure (Figure 2). Additional percutaneous transluminal angioplasty was performed due to the low ankle–brachial index and clinical evidence of poor perfusion. After seven days of empirical antibiotics, deep-tissue cultures yielded Bacteroides fragilis and Proteus mirabilis, both susceptible to ampicillin-sulbactam; the regimen was de-escalated to ampicillin-sulbactam for an additional 8 days in consultation with infectious diseases.

Free flap reconstruction was considered due to bone and tendon exposure but deferred owing to significant comorbidities and high surgical risk. Instead, NPWTi-d was initiated with reticulated foam dressings (Table 1), resulting in >70% granulation coverage of the wound, including previously exposed bone and tendon.

**Table 1:** NPWTi-d parameters applied in this case.

Parameter	Setting
Instillation solution	Normal saline
Soak time	10 min
Therapy (V.A.C.) time	2 h
Negative pressure	−125 mmHg
Number of cycles	4
Duration per cycle	7 days (before dressing change)

**Figure 3:**
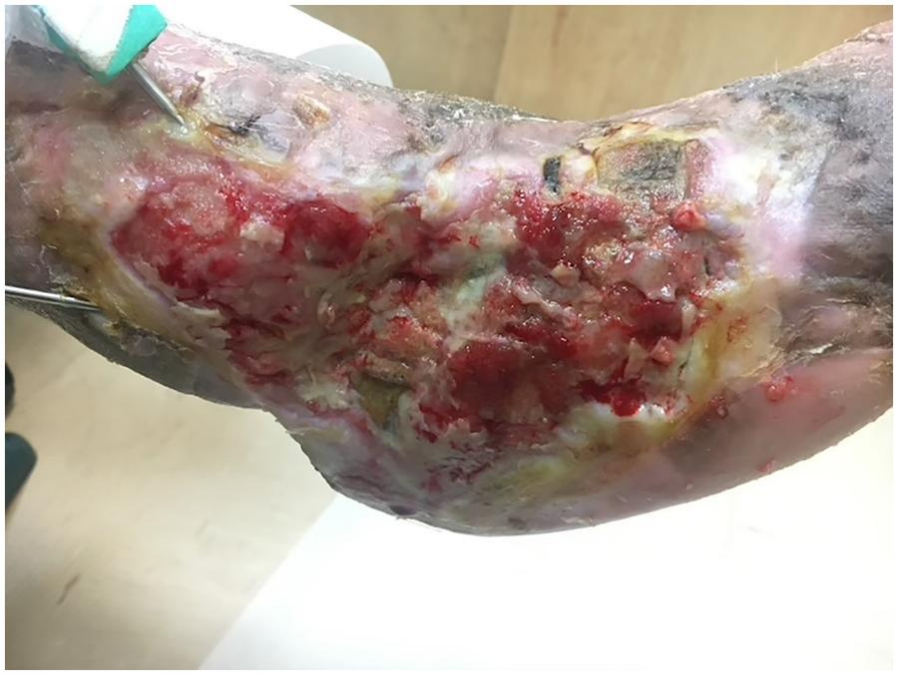
Wound status after staged multimodal therapies, including NPWTi-d followed by dermal substitute with conventional NPWT, showing near-complete granulation tissue coverage at three months with minimal residual tendon or bone exposure. A Kirschner wire was placed to maintain ankle and forefoot alignment during the healing process.

Subsequently, a bilayer artificial dermis composed of porcine atelocollagen with a fenestrated silicone outer membrane (Terudermis) with NPWT was applied to promote epithelialization and wound closure. Concurrent multidisciplinary care was optimized management of diabetes, ESRD, and PAOD. After three months, nearly complete granulation coverage was achieved with minimal residual tendon or bone exposure (Figure 3).

Functionally, as granulation progressed, the patient ambulated with crutches and performed most activities of daily living independently; pain during NPWTi-d, dermal substitute therapy, and subsequent rehabilitation was minimal (Visual Analog Scale 0–1/10), facilitating adherence. Through month 5 of follow-up, before death from three-vessel coronary artery disease with cardiogenic shock unrelated to the wound, there was no recurrence of infection, no new ulceration, and no need for further procedures based on treating-team documentation without additional wound images; major amputation was avoided. A structured clinical timeline is provided (Supplementary Table S2).

## Discussion

NF requires urgent surgical intervention, as broad-spectrum antibiotics alone are insufficient due to poor penetration in ischemic tissues (7). Delays beyond 24 h have been associated with up to a 9.4-fold increase in mortality (19). Debridement, fasciotomy, or necrosectomy often require multiple sessions, with an average of 4.8 procedures for wound bed preparation (20). These interventions frequently result in large defects with exposed bone or tendon.

In high-risk patients, wound healing is further impaired by comorbidities such as PAOD, ESRD, diabetes, and smoking, which compromise perfusion and tissue regeneration (21, 22). Amputation is often necessary in cases with extensive necrosis, deep muscle involvement, American Society of Anesthesiologists scores ≥3, or septic shock, with rates reaching 72.4% in DFU-associated NF (7, 9). While amputation may reduce surgical burden and control infection, it significantly impacts quality of life, underscoring the importance of alternatives that support infection control and tissue repair. Although free flap reconstruction is standard for wounds with exposure of bone, tendon, or muscle following extensive debridement (23, 24), comorbidities such as ESRD, PAOD, and smoking, as exemplified in this case, elevate perioperative risk and limit feasibility, necessitating alternative limb-salvage strategies.

NPWT has been shown to enhance granulation through improved perfusion, edema control, and cellular stimulation (25). NPWTi-d builds on these effects by adding cyclic instillation and dwell time, improving wound cleansing and mimicking debridement. Relative to standard NPWT or conventional dressings, meta-analytic data indicate higher odds of wound closure and bacterial count reduction, with fewer debridements and shorter therapy using NPWTi-d, supporting its prioritization in high-risk infected wounds (13). In this case, NPWTi-d was instrumental in managing a large, complex wound with bone and tendon exposure, promoting granulation while minimizing dressing frequency and patient discomfort. Normal saline was selected as the instillation fluid for its superior biocompatibility and comparable clinical outcomes relative to antiseptics (26).

Despite requiring inpatient care and technical expertise, NPWTi-d was prioritized due to the patient's comorbidities, anesthetic risk, and preference to avoid further surgery, with the goal of downstaging the reconstructive ladder and preventing flap reconstruction or amputation. While existing reports support NPWTi-d in postoperative NF and suggest benefits such as enhanced healing, bacterial reduction, and fewer complications (18), to our knowledge, no cases have described its use in wounds with extensive tendon and bone exposure in the context of multiple systemic comorbidities, as illustrated in this patient.

Dermal substitutes are frequently used for full-thickness skin defects, but their avascular nature limits early nutrient and oxygen delivery (27). Combining dermal substitutes with NPWT can overcome these limitations by reducing exudate, minimizing bacterial load, and enhancing angiogenesis and cellular infiltration. This approach is particularly beneficial in wounds with bone or tendon exposure, where studies have reported improved healing and shorter hospital stays (28).

We selected a bilayer porcine atelocollagen–silicone dermal substitute (Terudermis) because it is available in our institution and supports neodermis formation over exposed tendon and bone while providing a temporary epidermal barrier. This choice is supported by reports of NPWT combined with Terudermis and subsequent split-thickness skin grafting for tendon- and bone-exposed wounds (29) and was preferable to free flap or extensive graft reconstruction in this frail, ischemic diabetic foot.

In necrotizing fasciitis, case reports support the use of dermal substitutes with NPWT for large post-debridement wounds involving critical structures (14–17). However, treatment success depends on prior infection control and wound bed optimization. In this case, NPWTi-d was first applied to reduce bioburden and promote granulation in a high-risk wound, effectively downstaging the reconstructive ladder and enabling successful dermal substitute integration.

Compared with prior NF series using NPWTi-d, where sufficient granulation occurred in 9–16 days (mean 12.5 days); closure was obtained by split-thickness skin graft (65.6%), primary suture (28.1%) and flap transplantation (6.3%) (18), our wound required NPWTi-d followed by dermal substitute plus conventional NPWT to approach near-complete coverage by three months. This longer timeline reflects unusually hostile biology and geometry (PAOD, ESRD, diabetes, smoking; large defect with substantial exposed bone and tendon) and our deliberate avoidance of repeat debridements, while still achieving limb preservation without additional debridement or flap reconstruction.

A review of the contemporary literature found no prior description of a staged NPWTi-d followed by a dermal substitute with NPWT to achieve near-complete granulation over extensive bone and tendon exposure in DFU-associated NF with severe systemic comorbidities, while avoiding further debridement. This underscores a reconstruction-sparing trajectory in a high-risk limb.

Practically, this device-based, parameterized sequence is scalable where microsurgical reconstruction is unavailable or contraindicated. A stepwise, staged multimodal limb salvage algorithm (Supplementary Table S3) that (1) prioritizes infection control and rapid wound-bed optimization via NPWTi-d (parameters summarized in Table 1), then (2) converts to dermal substitutes plus NPWT to generate a vascularized neodermis over critical structures, provides a reconstruction-sparing pathway to limb salvage that aligns with international guidance emphasizing multidisciplinary, staged care in NF (30) and is particularly relevant in resource-limited settings given the high major-amputation burden of DFU-associated NF.

The patient prioritized limb preservation over additional operations and described a low treatment burden, reporting acceptable comfort with NPWTi-d and rehabilitation. She valued the cosmetic improvement as granulation matured and noted that the regimen remained compatible with daily routines, including hemodialysis scheduling.

The 2018 World Society of Emergency Surgery/Surgical Infection Society–Europe guidelines highlight the value of a multidisciplinary approach in necrotizing fasciitis, integrating early diagnosis, surgical intervention, infection control, and coordinated reconstruction planning (30). In this case, effective collaboration among cardiology, nephrology, infectious diseases, endocrinology, and plastic surgery enabled comprehensive systemic management and optimized wound healing, reinforcing the essential role of multidisciplinary care in high-risk NF cases.

Limitations include the single-case design, absence of functional follow-up beyond 3 months due to cardiovascular death at month 5, and restriction of vascular assessment to a single ankle–brachial index without toe pressures or duplex imaging. Endpoint heterogeneity with prior series limits time-to-closure comparisons. Wound status at month 5 lacked photographic confirmation and relied on treating-team documentation. This device-intensive strategy depends on access to NPWTi-d, dermal substitutes, and conventional NPWT, which may limit applicability in some low-resource settings.

## Conclusion

This case demonstrates that a staged, multimodal treatment strategy combining NPWTi-d and a dermal substitute under NPWT, supported by multidisciplinary coordination, can serve as an effective limb salvage approach for diabetic foot ulcer–associated necrotizing fasciitis with extensive tendon and bone exposure. By prioritizing infection control, granulation promotion, and individualized planning, this strategy optimized wound healing, avoided major reconstruction and amputation, and may extend current evidence on NPWT and dermal substitutes to similar high-risk cases. Clinical benefit extended beyond wound metrics, with assisted ambulation, minimal pain, and a stable wound through month 3 on direct follow-up and through month 5 as documented by the treating team, without recurrence or further procedures, although these observations are limited by the single-case design.

## Data Availability

The original contributions presented in the study are included in the article/Supplementary Material, further inquiries can be directed to the corresponding author.
