# Supplementary material for: Case Report: Negative pressure wound therapy with instillation and dwell time as adjuvant therapy for limb salvage in a complicated necrotizing fasciitis on ischemic diabetic foot
Source: Front Surg. 2026 Feb 5;13:1687275. doi: 10.3389/fsurg.2026.1687275 (PMC12916608; doi:10.3389/fsurg.2026.1687275)
Supplement: Supplementary file 1 [file Table1.docx]

Supplementary Table S1. Summary of the patient’s laboratory parameters upon hospital presentation

| Laboratory Parameter | Value | Unit |
| --- | --- | --- |
| White blood cell count | 14.5 | 10³/mm³ |
| Neutrophil | 89.1 | % |
| Hemoglobin | 9.5 | g/dL |
| C-reactive protein | 16.8 | mg/dL |
| Serum creatinine | 4.28 | mg/dL |
| Serum sodium | 133 | mEq/L |
| Serum potassium | 3.7 | mEq/L |
| Serum glucose | 230 | mg/dL |
