# Supplementary material for: Case Report: Negative pressure wound therapy with instillation and dwell time as adjuvant therapy for limb salvage in a complicated necrotizing fasciitis on ischemic diabetic foot
Source: Front Surg. 2026 Feb 5;13:1687275. doi: 10.3389/fsurg.2026.1687275 (PMC12916608; doi:10.3389/fsurg.2026.1687275)
Supplement: Supplementary file 2 [file Table2.docx]

Supplementary Table S2. Clinical timeline of key interventions, antibiotics, and wound outcomes

| Timepoint | Intervention / Procedure | NPWTi-d / Dermal substitute details | Antimicrobial therapy | Microbiology | Wound outcome / Milestones |
| --- | --- | --- | --- | --- | --- |
| Initial presentation | Urgent extensive debridement, fasciotomy, sequestrectomy | - | Empiric: teicoplanin + piperacillin–tazobactam + clindamycin (first 7 days) | - | Defect 250 cm²; bone exposure 95 cm²; tendon exposure |
| Vascular optimization | Percutaneous transluminal angioplasty (PTA) for poor perfusion | NPWTi-d initiated | Continue empiric regimen to day 7 | - | Hemodynamics/perfusion optimized  Start granulation |
| End of week 1 | - | First cycle of NPWTi-d (parameters summarized in Table 1) | De-escalation to ampicillin–sulbactam (additional 8 days) | Deep-tissue cultures: Bacteroides fragilis, Proteus mirabilis; both susceptible to ampicillin–sulbactam | Early granulation evident |
| Weeks 2–4 | NPWTi-d cycles ×3 (weekly) | NPWTi-d continued | Completed targeted course | - | >70% granulation, including over prior bone/tendon exposure |
| Weeks 5–12 | Dermal substitute plus conventional NPWT | Dermal substitute in situ; NPWT to support neodermis | - | - | Progressive granulation; near-complete coverage by month 3 |
| Month 5 follow-up | - | - | - | - | Wound stable; no recurrence, no new ulceration, no further procedures; major amputation avoided; patient expired from 3-vessel CAD (non-wound cause) |
