# Supplementary material for: Case Report: Negative pressure wound therapy with instillation and dwell time as adjuvant therapy for limb salvage in a complicated necrotizing fasciitis on ischemic diabetic foot
Source: Front Surg. 2026 Feb 5;13:1687275. doi: 10.3389/fsurg.2026.1687275 (PMC12916608; doi:10.3389/fsurg.2026.1687275)
Supplement: Supplementary file 3 [file Table3.docx]

Supplementary Table S3. Staged multimodal limb salvage algorithm used in this case.

| Stage | Clinical situation / trigger | Key local wound management | Systemic / functional management | Primary goal |
| --- | --- | --- | --- | --- |
| Stage 1. Emergency source control and systemic optimization | Presentation with necrotizing fasciitis on an ischemic diabetic foot, systemic sepsis, soft-tissue necrosis | Emergency radical debridement and fasciotomy, removal of necrotic and devitalized tissue, wound left open for reassessment | Broad-spectrum intravenous antibiotics; hemodynamic resuscitation; glycemic control; early vascular, cardiology, and nephrology co-management to address underlying ischemia and organ dysfunction | Immediate life- and limb-saving infection source control and optimization of systemic and vascular status to support subsequent wound healing |
| Stage 2. NPWTi-d–based bioburden control and granulation | Wound has been adequately debrided but remains large, deep, and contaminated, with exposed tendon and bone after initial emergency surgery | Initiation of NPWTi-d using normal saline instillation with standardized pressure and dwell parameters after thorough debridement; only limited bedside debridement/trimming as needed, aiming to reduce the need for further operative debridement; promotion of robust granulation over exposed critical structures | Continuation of targeted antibiotic therapy and systemic optimization; gradual introduction of protected weight bearing with assistive devices and pressure off-loading | Reduce bioburden and edema, decrease the need for repeated operative debridement, and induce healthy granulation tissue covering tendon and bone to prepare the wound for dermal regeneration |
| Stage 3. Dermal regeneration with dermal substitute + NPWT | Infection is controlled and the wound is clean, but dermal tissue remains insufficient and some critical structures are still partially exposed | Application of a bilayer porcine atelocollagen–silicone dermal substitute (Terudermis) firmly secured to the wound bed and supported by conventional NPWT | Ongoing medical optimization; continued protected weight bearing and off-loading | Generate a vascularized neodermis over previously exposed tendon and bone and create a stable, pliable wound bed suitable for epithelialization or potential grafting if required |
| Stage 4. Consolidation, epithelialization, and rehabilitation | Neodermis formed with near-complete granulation, no recurrent infection, and a frail patient with high operative risk | Transition from NPWT to appropriate dressings as the wound epithelializes; continued local wound care without major flap or extensive split-thickness skin graft | Progression of assisted ambulation, functional rehabilitation, long-term off-loading strategies, and optimization of diabetic and cardiovascular risk factors | Achieve durable limb preservation without major amputation, with acceptable pain control and functional mobility in a medically complex patient |
